# Preparation, Characterization, and Antimicrobial Activities of Bimetallic Complexes of Sarcosine with Zn(II) and Sn(IV)

**DOI:** 10.1155/2013/351262

**Published:** 2013-10-22

**Authors:** Yasir Arafat, Saqib Ali, Saira Shahzadi, Muhammad Shahid

**Affiliations:** ^1^Department of Chemistry, GC University, Faisalabad 38000, Pakistan; ^2^Department of Chemistry, Quaid-i-Azam University, Islamabad 45320, Pakistan; ^3^Department of Chemistry and Biochemistry, University of Agriculture, Faisalabad 38000, Pakistan

## Abstract

Heterobimetallic complexes of Zn(II) and Sn(IV) with sarcosine have been synthesized at room temperature under stirring conditions by the reaction of sarcosine and zinc acetate in 2 : 1 molar ratio followed by the stepwise addition of CS_2_ and organotin(IV) halides, where R = Me, *n-*Bu, and Ph. The complexes were characterized by elemental analysis, FT-IR and NMR (^1^H, ^13^C) spectroscopy. IR data showed that the ligand acts in a bidentate manner. NMR data revealed the four coordinate geometry in solution state. *In vitro* antimicrobial activities data showed that complexes (**3**) and (**4**) were effective against bacterial and fungal strains with few exceptions.

## 1. Introduction

Organotin compounds are amongst the most widely used organometallic compounds. Over the last several decades, they have been utilized for a variety of industrial and agricultural applications including pesticides, fungicide, and antifouling agents [1]. In general, the biochemical activity of organotin(IV) carboxylates is greatly influenced by the structure of the molecule and the coordination number of the tin atom [2, 3]. Therefore, the recognition of the importance between the biological properties and the structure of organotin(IV) carboxylates [4] has stimulated the study of carboxylates of tin.

The diverse structural motifs are known in organotin compounds and attributed to the ambidentate character of the carboxylate ligands [5]. Steric and electronic attributes of organic substituents on tin and/or the carboxylate moiety impart significant influence on the structural characteristics in tin carboxylates. Therefore, synthesis of new organotin carboxylates with different structural features will be beneficial in the development of pharmaceutical organotin and in other properties and applications. 

Meanwhile, dithiocarbamate (DTC) is the ligand which strongly bonds with metal ions and can stabilize metal complexes with high oxidation number [6]. Organotin(IV) dithiocarbamate complexes have been widely studied due to Sn-S bond in their structure and the effects of the bonding on diversified applications basically in biological field [7, 8].

Zinc chloride is chiefly used as a catalyst for the Fischer indole synthesis and Friedel-Craft acylation reaction that was involved in the synthesis of organic  compounds used in the laboratory [9]. The medical applications of Zn(II) compounds include treatments of parasitic diseases (eczema, ringworm, fungus, and athletes foot), and in many biological processes zinc plays an important role as metalloenzymes in which Zn(II) is coordinated by a ligand that leads to a structural as well as functional model [10].

**Figure 1:**
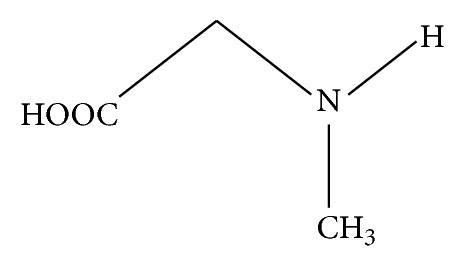
Chemical structure of sarcosine.

In our previous work, we reported several organotin complexes with oxygen and sulfur donor atoms [11–15]. As an extension of this research program, we report here the complexes of sarcosine (Figure 1) containing Zn(II) and Sn(IV) to study the effect of Zn along with tin on biological activities and compare these results with already reported organotin complexes of sarcosine [16–18]. 

These complexes were characterized by elemental analysis, IR and multinuclear NMR (^1^H, ^13^C). These were also examined to check their antibacterial and antifungal activity *in vitro*.

## 2. Experimental

### 2.1. Materials and Methods

N-Methyl glycine (sarcosine), zinc acetate, carbon disulfide, and organotin(IV) halides were of Sigma-Aldrich (UK) origin. The organic solvents (chloroform, *n*-hexane, ethanol, methanol, DMSO, and acetone) were procured from Merck (Germany). The solvents were dried [19] prior to use. Nutrient broth, nutrient agar, and potato dextrose agar were of oxide (UK) origin. Autoclave and laminar air flow were purchased from Omron and Dalton companies of Japan, respectively. Micropipettes were purchased from Gilson (France). The melting points were determined on an electrothermal melting point apparatus, model Staurt SMP3 by using capillary tubes. Elemental analysis was done for carbon, hydrogen, nitrogen, and sulphur on a CHNS-932 elemental analyzer Leco Corporation (USA). The infrared spectra were recorded as KBr pellets on Perkin Elmer 1000 spectrometer in the frequency range of 4000–250 cm^−1^. NMR (^1^H and ^13^C) spectra were recorded on Bruker AM-300 MHz FT-NMR spectrometer (Germany) using CDCl_3_ as an internal reference. 

### 2.2. General Procedure for the Synthesis of Complexes

**Scheme 1:**
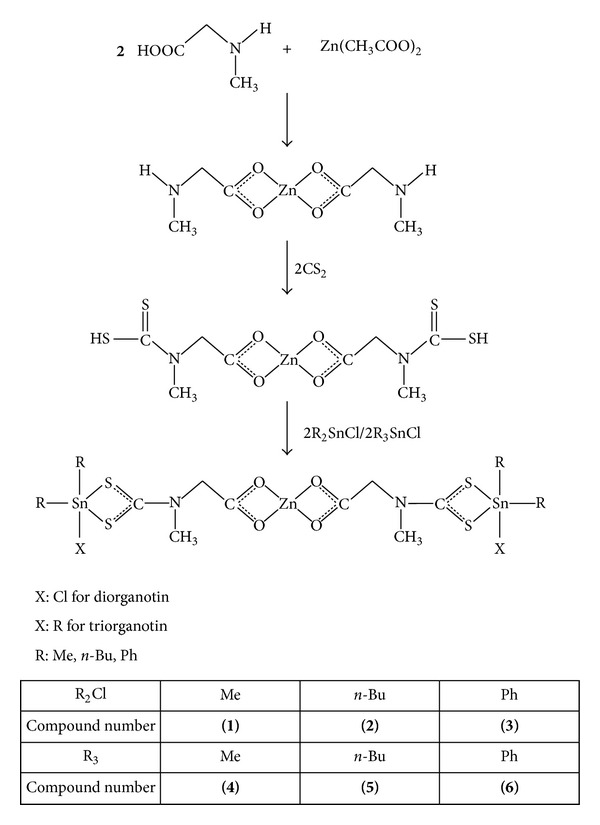


Sarcosine (0.178 g, 2 mmol) was dissolved in methanol (25 mL) in a round bottom flask with continuous stirring at room temperature. Then solution of zinc acetate (0.22 g, 1 mmol) in methanol (3 mL) was added dropwise to the above solution, and mixture was stirred continuously for 20 minutes. After that CS_2_ (0.120 mL, 2.0 mmol) in methanol (5 mL) was added dropwise to the reaction mixture and stirred for 0.5 hr at room temperature. Subsequently, the solution of diorganotin dichloride/triorganotin chloride (2 mmol) in methanol (50 mL) was added and the mixture was continuously stirred for 4 hr. Solvent was slowly evaporated at room temperature and product obtained was dried in air. Purity was checked by TLC and recrystallization was done in methanol : *n*-hexane (1 : 1) (see Scheme 1).

## 3. Results and Discussion

The ligand and synthesized complexes are solid and stable in air. They have sharp melting points. Elemental analysis was done to compare the observed values with predicted values of percentage of the carbon, hydrogen, nitrogen, and sulfur. The physical data of ligand and synthesized complexes is summarized in Table 1.

### 3.1. Infrared Spectroscopy

IR spectra of the ligand and its newly synthesized complexes (**1**)–(**6**) were recorded as KBr pellets in the range of 4000–250 cm^−1^. The characteristic infrared absorption frequencies (cm^−1^) have been listed in Table 2.

The IR spectrum of the ligand exhibited a band at 3365 cm^−1^, which was attributed to the NH stretching mode. Deprotonation of carboxylic group is confirmed by the absence of OH band in the spectra of the complexes. Regarding the carboxyl vibrational modes, there are different binding possibilities [20, 21]:

1. bridged structure, *ν*_*s*_(COO) = 1560–1540 cm^−1^,
2. dimer and monomer chelate structures, *ν*_*a*_(COO) = 1640–1560 cm^−1^,
3. “free ester” monodentate structure, **ν**(C–O) = 1680–1640 cm^−1^ and **ν**(C=O) = 1770 1700 cm^−1^.

The carboxylate group gives strong asymmetric and symmetric stretching bands in the range of 1602–1650 cm^−1^ and 1433–1470 cm^−1^, respectively, and it is assignable to a chelate or monodentate structure; however, the absence of **ν**C=O band in expected range is indicative of the presence of the chelate form. Bidentate nature of ligand is also confirmed by Δ**ν**COO value that is less than 200 cm^−1^ in all complexes [22]. Presence of **ν**Zn–O vibrational bands in the range of 310–385 cm^−1^ in all the complexes [23] confirms the zinc carboxylate interaction. It has been reported [15] that the observation of a single **ν**C=S absorption in the region around 1000 cm^−1^ is indicative of dithiocarbamate groups that are bonded symmetrically or bidentate in nature. A strong absorption band of **ν**(C=S) lies in the range of 1013–1043 cm^−1^, while **ν**(C–S) band appears in the lower frequency range, 942–988 cm^−1^ [24]. The Sn–S bond formation is confirmed by the appearance of a new band in the range of 456–480 cm^−1^ [25]. The strong band at 302–318 cm^−1^ was assigned to **ν**(Sn–Cl) absorption. The Sn–C band was observed in the range of 530–547 cm^−1^ for complexes (**1**) and (**2**)–(**5**) but at 272 and 280 cm^−1^ for complexes (**3**) and (**4**), respectively, which are di- and triphenyltin derivatives. The absence of the band in the range 440–420 cm^−1^ which is commonly assigned to the **ν**(Sn–N) mode in analogous compounds [26, 27] rules out amino coordination to tin, thus leading support to the proposed carboxylate carboxyl atom [16] and dithiolate sulphur atom coordination in all synthesized complexes. 

### 3.2. ^1^H NMR Spectroscopy

^1^H NMR spectra of the ligand and complexes (**1**)–(**6**) were recorded in deuterated DMSO to find the behaviour of magnetically nonequivalent protons. The data is presented in Table 3. 

The number of protons calculated by integration of peaks is in excellent agreement with those theoretically calculated by incremental method [28]. The absence of OH signal in the complex suggested the deprotonation of carboxylic acid group for O → Zn coordination through COO^−^ anions. Two resonance signals appeared as a singlet at 3.15 ppm and 2.48 ppm for the methylene and methyl protons, respectively, in the free ligand. These signals exhibited downfield shift to 3.63–3.99 ppm and 2.79–2.96 ppm in the complexes (**1**)–(**6**) for methylene and methyl protons, respectively.

The CH_3_ protons in compound (**1**) appear as a singlet at 1.27 ppm with tin satellites having ^2^ *J*[^119^Sn–^1^H] = 96 Hz. In compound (**2**), the protons of butyl group appear as a multiplet in the region 0.88–0.93 ppm. The terminal CH_3_ group of butyl gives triplet at 0.23 ppm with ^3^ *J*[^1^H–^1^H] value of 7.2 Hz. The proton chemical shift of the methyl group in compound (**3**), attached to the Sn, gives a singlet at 0.51 ppm. The phenyl moieties of di- and triphenyltin(IV) derivatives show a complex pattern and were assigned according to the literature [29]. The ^2^ *J*[^119^Sn–^1^H] value for di- and triphenyltin(IV) derivatives is 82 Hz, in the range expected for pentacoordinated tin atom, and consistent with C–Sn–C angle of 126.5°.

### 3.3. ^13^C NMR

The assignments of –CSS group in investigated compounds is straightforward which are observed in the range of 202.5–202.8 ppm indicating the coordination of sulfur to the tin atom.

Table 4 lists the chemical shifts of ^13^C and tin-carbon coupling constants for the reported complexes (**1**)–(**6**). The ^13^C NMR chemical shifts due to the phenyl groups are observed at positions comparable to other similar compounds [30, 31]. The ^13^C NMR chemical shift due to –COO carbon atom is observed at 179.2 ppm in the free ligand while in the complexes it exhibits downfield shift in the range 181.9–186.3 ppm. Coordination of the tin atom in di- and triorganotin has been related to ^*n*^ *J*(^119^Sn–^13^C) coupling constants. The ^*n*^ *J*(^119^Sn–^13^C) coupling for complexes (**1**) and (**6**) is 397 and 640 Hz, respectively, which is indicative of four coordinate geometry [30] in solution state. 

### 3.4. Antibacterial Activity

Sarcosine and synthesized complexes (**1**)–(**6**) were tested *in vitro* for their antibacterial activity against gram positive and gram negative bacterial strains including* B. subtilis* and *S. aureus* and *P. multocida* and *E. coli*, respectively, and the results are given in Table 5. Ampicillin was used as a standard drug. The results revealed that all the newly synthesized complexes show higher activity than the ligand but markedly lower than the standard drug with few exceptions. 

Complex (**5**) showed maximum inhibitory action against *B. subtilis* (54.5 ± 0.86) and *E. coli* (51.0 ± 1.00), probably more than standard drug (40.00 ± 1.41) and (39.5 ± 0.86), respectively. Complex (**2**) displayed lowest inhibition zone in case of *B. subtilis* (20.5 ± 0.86) and* S. aureus* (21.0 ± 1.00). The ligand exhibited low activities, while the complexes exhibited moderate activities as compared to standard drug towards all the bacterial strains. Complex (**6**) showed moderate antibacterial activity against all the bacterial strains. Among all the complexes, the tributyltin complex (**5**) showed significant antibacterial activity. It was clear from the data that antimicrobial activities varied according to substitution (increase in substitution on the Sn(IV) enhances the antimicrobial activity) [32].

Data revealed that the synthesized complexes showed more activity against gram positive bacterial strain than gram negative strains. It was due to the difference in the composition of cell wall of both the gram positive and gram negative strains [33]. It was also suggested that the anti-microbial activity of the complexes was either due to killing of microbes or inhibiting their reproduction by blocking their active sites [34]. 

The results show that all compounds exhibit antibacterial activity, and in many cases, complexes are more potent in their inhibition properties than the free ligand. This can be explained in terms of the greater lipid solubility and cellular penetration of the complexes [35]. It is clear that the coordination enhances the antibacterial activity and clearly indicates that the newly synthesized complexes in the present studies are more active against gram positive than gram negative bacteria. The preliminary results achieved have led us to conclude that these types of complexes should be studied in detail for their applications in diverse area.

The screening data of a particular ligand and its metal complexes show that the former has greater activity than the latter from the biochemical point of view. On comparing the results in general, it may be concluded that complexes containing Zn(II) and Sn(IV) have greater inhibiting power than the free ligands as compared to organotin complexes with sarcosine [16, 17] against all the microbes. 

### 3.5. Antifungal Activity

The synthesized compounds (**1**)–(**6**) and sarcosine were tested for antifungal activity by using fluconazole as standard drug against four fungal strains including *A. flavus, A. niger, A. alternate, and G. lucidum* by disc diffusion method [36]. The results are summarized in Table 6. It was evident from tabulated data that the sarcosine as well as the synthesized complexes exhibited, variety of fungicidal activity. Sarcosine exhibited maximum activity against* A. flavus *(37.5 ± 0.86) while complex (**5**) exhibited maximum activity (39.5 ± 1.65) against* G. lucidum*. Complex (**2**) showed the lowest inhibition zone (19.0 ± 1.00) against *G. lucidum*. It is noted that tributyltin complexes showed the greatest inhibitory effect against fungi as compared to other alkyl groups. Thus the presence of butyl groups in compound (**5**) bonded with tin atom was responsible for the rise of antifungal activity [37]. The increased activity of complexes might be due to the coordination of zinc and tin with oxygen and sulfur, respectively [38]. From the data it might be concluded that triorganotin compounds were usually more effective against the fungi than diorganotin compounds [39] in contrast to earlier reported compounds in the literature [16]. This might be due to the presence of Zn(II) along with Sn(IV) which enhances the fungicidal activity of triorganotin complexes.

### 3.6. Structure Activity Relationship

Although it is difficult to make out an exact structure-activity relationship between the antimicrobial activity and the structure of these complexes, it can possibly be concluded that the chelation as well as the addition of a substrate enhances the activity of the complexes. The variation in the toxicity of different antibacterial agents against various organisms depends on either the impermeability of the cell or differences in site of action or ability to cause mutations in the microorganism. Though the results suggest that the ligand has a remarkable toxic property, their complexes of tin inhibit the growth of microorganisms to a greater extent. This is in accordance with earlier reports [40]. Further, the greater activity of the complexes can also be explained on the basis of their higher solubility of the particles.

## 4. Conclusion

The FT-IR data of synthesized complexes clearly demonstrate that the zinc and tin become attached with the oxygen and sulphur of the ligand in a bidentate mode. In solid state, chlorodiorganotin complexes exhibit the penta/hexacoordinated geometry, whereas the triorganotin(IV) complexes show the five-coordinate geometry. Biological activity data showed that all the complexes were biologically active with few exceptions. These complexes were found to be more potent inhibitors toward fungal culture as compared to bacterial strains. 

## Figures and Tables

**Table 1:** Physical data of heterobimetallic complexes with sarcosine.

Comp. no.	Molecular formula	Mol. weight	% yield	m.p (°C)	Elemental analysis			
					% C Calcd. (found)	% H Calcd. (found)	% N Calcd. (found)	% S Calcd. (found)
HL	C_3_H_7_O_2_N	89.09	—	208	40.45	7.92	15.71	—
					(40.42)	(7.95)	(15.68)	
(**1**)	C_12_H_22_O_4_N_2_S_4_Cl_2_ZnSn_2_	760.31	75	121-122	18.95	2.91	3.68	16.87
					(18.99)	(2.95)	(3.64)	(16.89)
(**2**)	C_24_H_46_O_4_N_2_S_4_Cl_2_ZnSn_2_	928.63	68	87–91	31.04	4.99	3.01	13.81
					(31.07)	(4.96)	(3.04)	(13.78)
(**3**)	C_32_H_30_O_4_N_2_S_4_Cl_2_ZnSn_2_	1008.53	57	107–109	38.10	2.99	2.77	12.71
					(39.12)	(2.96)	(2.74)	(12.74)
(**4**)	C_14_H_28_O_4_N_2_S_4_ZnSn_2_	719.61	67	145-146	23.36	3.92	3.89	17.82
					(23.39)	(3.96)	(3.92)	(17.78)
(**5**)	C_32_H_64_O_4_N_2_S_4_ZnSn_2_	971.53	51	224-225	39.55	6.64	2.88	13.20
					(39.50)	(6.68)	(2.85)	(13.17)
(**6**)	C_44_H_40_O_4_N_2_S_4_ZnSn_2_	1091.89	73	86–88	48.39	3.69	2.56	11.74
					(48.36)	(3.65)	(2.59)	(11.78)

**Table 2:** IR spectral data (cm^−1^) of heterobimetallic complexes with sarcosine.

Comp no.	**ν**(NH)	**ν**(COO)		Δ*ν*	**ν**(C=S)	**ν**(C–S)	**ν**(Sn–C)	**ν**(Sn–S)	**ν**(Sn–Cl)	**ν**(Zn–O)
		**ν**(COO) (asym)	**ν**(COO) (sym)							
HL	3365	1621	1407	214	—	—	—	—	—	—
(**1**)	—	1642	1462	180	1033	955	526	461	311	364
(**2**)	—	1618	1433	185	1013	942	530	472	302	352
(**3**)	—	1602	1440	162	1025	988	272	466	318	347
(**4**)	—	1622	1456	166	1023	938	538	485	—	371
(**5**)	—	1635	1468	167	1043	965	547	456	—	385
(**6**)	—	1650	1470	180	1030	970	280	480	—	310

**Table 3:** ^1^H NMR data^a–d^ (ppm) of heterobimetallic complexes with sarcosine.

Proton no.	HL	(**1**)	(**2**)	(**3**)	(**4**)	(**5**)	(**6**)
2, 2′	3.15s	3.97s	3.99s	3.88s	3.74s	3.63s	3.95s
3, 3′	2.48s	2.89s	2.80s	2.96s	2.85s	2.94s	2.79s

^a^Compound (**1**) Sn-CH_3_Cl, 1.27s ^2^ *J*[96]; compound (**2**) Sn-CH_2_CH_2_CH_2_CH_3_Cl, 0.88–0.93m, 0.23t (7.2); compound (**3**) Sn-C_6_H_5_Cl, 7.91d ^2^ *J*[82], 7.50–7.53m, 7.42–7.50m; compound (**4**) Sn-CH_3_, 0.51s ^2^ *J*[82]; compound (**5**) Sn-CH_2_CH_2_CH_2_CH_3_, 0.64–1.1m, 0.23t (7.2); compound (**6**) Sn-C_6_H_5_, 7.91d ^2^ *J*[82], 7.50–7.53m, 7.42–7.50m.

^b^Multiplicity is given as s: singlet, d: doublet, t: triplet, m: multiplet.

^c^Coupling constant, ^*n*^ *J*[^119^Sn, ^1^H] and ^*n*^ *J*[^1^H, ^1^H] in Hz are given in square bracket and parenthesis, respectively.

^d^

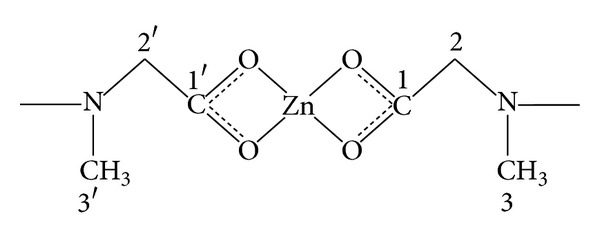

**Table 4:** ^
13^C NMR data^a,b^ (ppm) of heterobimetallic Zn(II) and Sn(IV) complexes with sarcosine.

Carbon	HL	(**1**)	(**2**)	(**3**)	(**4**)	(**5**)	(**6**)
1, 1′	179.2	186.3	181.9	183.7	185.4	184.3	183.8
2, 2′	56.6	56.8	56.3	56.9	56.7	56.6	56.8
3, 3′	41.5	41.3	41.3	41.5	41.4	41.2	41.5
–CSS	—	202.6	202.5	202.8	201.7	202.8	202.7

^a^Compound (**1**) Sn-CH_3_Cl, (C-*α*) 2.1 ^1^ *J*[397]; compound (**2**) Sn-CH_2_CH_2_CH_2_CH_3_Cl, (C-*α*) 29.5, (C-*β*) 27.4, (C-*γ*) 26.2, (C-*δ*) 13.9; compound (**3**) Sn-C_6_H_5_Cl, (C-*α*) 137.6 ^1^ *J*[640], (C-*β*) 132.8 ^2^ *J*[49.0], (C-*γ*) 129.9, (C-*δ*) 124.8; compound (**4**) Sn-CH_3_, (C-*α*) 2.3 ^1^ *J*[396]; compound (**5**) Sn-CH_2_CH_2_CH_2_CH_3_, (C-*α*) 31.1 [349], (C-*β*) 27.1 ^2^ *J*[21], (C-*γ*) 28.2 ^3^ *J*[63], (C-*δ*) 14.1; compound (**6**) Sn-C_6_H_5_, (C-*α*) 138.0 ^1^ *J*[640], (C-*β*) 133.0 ^2^ *J*[49.0], (C-*γ*) 130.0, (C-*δ*) 125.1.

^b^Chemical shifts (*δ*) in ppm: ^*n*^ *J*[^119^Sn, ^13^C] in Hz is listed in parenthesis.

**Table 5:** Antibacterial activity data^a–e^ of complexes and ligand against selected bacterial strains.

Compound no.	Bacterial inhibition zone (mm)			
	*S. aureus *	*B. subtilis *	*E. coli *	*P. multocida *
HL	12.5^c^ ± 0.86	13.0^c^ ± 1.00	12.5^c^ ± 0.86	12.0^c^ ± 1.41
(**1**)	20.8^bc^ ± 1.00	18.0^c^ ± 1.00	25.2^c^ ± 0.86	21.0^ab^ ± 1.00
(**2**)	21.0^bc^ ± 1.00	20.5^bc^ ± 0.86	27.5^bc^ ± 0.86	24.5^ab^ ± 0.86
(**3**)	20.8^bc^ ± 1.00	18.6^c^ ± 1.00	26.2^c^ ± 0.86	21.3^ab^ ± 1.00
(**4**)	30.0^ab^ ± 1.41	21.5^bc^ ± 1.65	28.0^bc^ ± 1.41	23.0^ab^ ± 1.00
(**5**)	32.5^ab^ ± 1.65	54.5^a^ ± 0.86	51.0^a^ ± 1.00	32.5^a^ ± 1.65
(**6**)	31.5^ab^ ± 0.86	33.0^bc^ ± 1.41	32.0^ab^ ± 1.00	30.0^ab^ ± 1.41
Ampicillin	38.5^a^ ± 0.86	40.0^ab^ ± 1.41	39.5^ab^ ± 0.86	32.0^ab^ ± 1.41

^a^Concentration: 1 mg/mL in DMSO.

^b^Standard: ampicillin.

^c^0: no activity, 5–10: activity present, 11–25: moderate activity, 26–40: strong activity.

^d^Antibacterial values are mean ± S.D of samples analyzed individually in triplicate at *P* < 0.1.

^e^Different letters in superscript indicate significant differences.

**Table 6:** Antifungal activity data^a–e^ of complexes and ligand against selected fungal strains.

Compound no.	Fungal inhibition zone (mm)			
	*A. alternata *	*G*. *lucidum *	*A. flavus *	*A*.* niger *
HL	27.0^ab^ ± 1.00	11.5^c^ ± 0.86	37.5^ab^ ± 0.86	14.5^bc^ ± 1.65
(**1**)	26.4^ab^ ± 1.00	19.3^bc^ ± 1.00	20.2^bc^ ± 1.65	20.3^ab^ ± 0.86
(**2**)	20.0^c^ ± 1.41	19.0^bc^ ± 1.00	20.5^bc^ ± 1.65	20.5^bc^ ± 0.86
(**3**)	26.9^bc^ ± 1.41	20.2^ab^ ± 1.65	35.2^bc^ ± 1.65	21.2^bc^ ± 0.86
(**4**)	30.0^ab^ ± 1.41	18.5^bc^ ± 0.86	19.5^c^ ± 1.65	18.5^bc^ ± 0.86
(**5**)	30.0^ab^ ± 1.41	39.5^ab^ ± 1.65	37.0^ab^ ± 1.65	33.5^ a^ ± 1.65
(**6**)	28.5^ab^ ± 0.86	25.0^bc^ ± 1.00	32.0^bc^ ± 1.41	19.5^bc^ ± 0.86
Fluconazole	30.5^a^ ± 0.86	41.5^a^ ± 0.86	54.0^a^ ± 1.41	12.5^c^ ± 0.86

^a^Concentration: 1 mg/mL in DMSO.

^b^Standard: fluconazole.

^c^0: no activity, 5–10: activity present, 11–25: moderate activity, 26–40: strong activity.

^d^Antibacterial values are mean ± S.D of samples analyzed individually in triplicate at *P* < 0.1.

^e^Different letters in superscript indicate significant differences.

## References

[1] Appel KE (2004). Organotin compounds: toxicokinetic aspects. *Drug Metabolism Reviews*.

[2] Molloy KC, Purcell TG, Hahn E, Schumann H, Zuckerman JJ (1986). Organotin biocides. 4. Crystal and molecular structure of tricyclohexylstannyl 3-indolylacetate, incorporating the first monodentate carboxylate group bonded to a triorganotin(IV). *Organometallics*.

[3] Holmes RR (1989). Organotin cluster chemistry. *Accounts of Chemical Research*.

[4] Barbieri R, Silvestri A, Giudice MTL, Ruisi G, Musmeci MT (1989). The binding of trialkyltin(IV) moieties to rat haemoglobin, and the structure of model systems, studied by tin-119 Mössbauer spectroscopy. *Journal of the Chemical Society, Dalton Transactions*.

[5] Sadiq-Ur-Rehman S, Shahid K, Ali S, Bhatti MH, Parvez M (2005). Organotin esterification of (*E*)-3-(3-fluoro-phenyl)-2-(4-chlorophenyl)-2-propenoic acid: synthesis, spectroscopic characterization and *in vitro* biological activities. Crystal structure of [Ph_3_Sn(OC(O)C(4-ClC_6_H_4_) = CH(3-FC_6_H_4_))]. *Journal of Organometallic Chemistry*.

[6] Heard PJ (2005). *Progress in Inorganic Chemistry*.

[7] Thoonen SHL, Deelman B, van Koten G (2004). Synthetic aspects of tetraorganotins and organotin(IV) halides. *Journal of Organometallic Chemistry*.

[8] Hussain H, Ahmad VU, Green IR, Krohn K, Hussain J, Badshah A (2007). Antibacterial organotin(IV) compounds, their synthesis and spectral characterization. *Arkivoc*.

[9] Dike SY, Merchant JR, Sapre NY (1991). A new and efficient general method for the synthesis of 2-spirobenzopyrans: first synthesis of cyclic analogues of precocene I and related compound. *Tetrahedron*.

[10] da Silva LE, de Sousa PT, Joussef AC, Piovezan C, Neves A (2008). Synthesis, structure and physicochemical properties of zinc and copper complexes based on sulfonamides containing 8-aminoquinoline ligands. *Química Nova*.

[11] Anwer J, Ali S, Shahzadi S, Shahid M, Sharma SK, Qanungo K (2013). Synthesis, characterization, semi-empirical study and biological activities of homobimetallic complexes of tranexamic acid with organotin(IV). *Journal of Coordination Chemistry*.

[12] Jabbar S, Shahzadi I, Rehman R (2012). Synthesis, characterization, semi-empirical study and biological activities of organotin(IV) complexes with cyclohexylcarbamodithioic acid as biological active ligand. *Journal of Coordination Chemistry*.

[13] Khan HN, Ali S, Shahzadi S, Helliwell M (2012). Synthesis, spectroscopy and antimicrobial activity of chloro-organotin(IV) complexes of S-donor ligand: crystal structure of chloro-t-dibutyltin[4-methyl-1-piperidine]thiocarboxylate. *Russian Journal of Inorganic Chemistry*.

[14] Shah FA, Ali A, Shahzadi S, Rizzoli C, Ahmad A (2012). Synthesis, spectral characterization and X-ray crystal structure of biologically active organotin(IV) 3-[(3′, 5′-dimethylphenylamido)]propanoates. *Journal of Iranian Chemical Society*.

[15] Amin MM, Ali S, Shahzadi S, Sharma SK, Qanungo K (2011). Di- and triorganotin(IV) complexes of 2-aminobenzoic acid with and without triphenylphosphine: synthesis, spectroscopy, semi-empirical study, and antimicrobial activities. *Journal of Coordination Chemistry*.

[16] Khoo E, Goh NK, Eng G, Whalen DJ, Hzell A (1995). Synthesis, characterization and fungicidal activity of triphenyl derivatives of sarcosine: crystal structures of [Ph_3_Sn(OCOCH_2_NH_2_CHB)_2_]Cl and [Ph_3_(OCOCH_2_NH_2_CH_3_)2]NCS. *Applied Organometallic Chemistry*.

[17] Kovala-Demertzi D, Tauridou P, Moukarika A, Tsangaris JM, Raptopoulou CP, Terzis A (1995). Synthesis and characterization of tin(IV) and organotin(IV) 1,4-dimethylpiperazine-2,5-dione (cyclosarcosylsarcosine) adducts. *Journal of the Chemical Society, Dalton Transactions*.

[18] Ronconi L, Marzano C, Russo U, Sitran S, Graziani R, Fregona D (2002). Synthesis, characterization and *in vitro* cytotoxicity of new organotin(IV) derivatives of N-methylglycine. *Journal of Inorganic Biochemistry*.

[19] Armarego WLF, Chai CLL (2000). *Purification of Laboratory Chemicals*.

[20] Honnick WD, Zuckerman JJ (1979). Diorganotin halide carboxylates, thiocarboxylates and halide haloacetates. *Journal of Organometallic Chemistry*.

[21] Tiekink ERT (1991). Structural chemistry of organotin carboxylates: a review of the crystallographic literature. *Applied Organometallic Chemistry*.

[22] Nath M, Jairath R, Eng G, Song X, Kumar A (2005). Synthesis, spectral characterization and biological studies of some organotin(IV) complexes of l-proline, *trans*-hydroxy-l-proline and l-glutamine. *Spectrochimica Acta A*.

[23] Hammes BS, Kieber-Emmons MT, Letizia JA (2003). Synthesis and characterization of several zinc(II) complexes containing the bulky heteroscorpionate ligand bis(5-tert-butyl-3-methylpyrazol-2-yl)acetate: relevance to the resting states of the zinc(II) enzymes thermolysin and carboxypeptidase A. *Inorganica Chimica Acta*.

[24] Singh R, Kaushik NK (2008). Spectral and thermal studies with anti-fungal aspects of some organotin(IV) complexes with nitrogen and sulphur donor ligands derived from 2-phenylethylamine. *Spectrochimica Acta A*.

[25] Singh HL, Singh JB (2012). Synthesis and characterization of new lead(II) and organotin(IV) complexes of schiff bases derived from histidine and methionine. *International Journal of Inorganic Chemistry*.

[26] Saxena A, Tandon JP, Molloy KC, Zuckerman JJ (1982). Tin(IV) complexes of tridentate schiff bases having ONS donor systems. *Inorganica Chimica Acta*.

[27] Saxena AK, Singh HB, Tandon JP (1980). Synthesis and characterization of tin(IV) complexes of azines. *Synthesis Reactivity Inorganic and Metal-Organic Chemistry*.

[28] Kalinowski HO, Berger S, Brown S (1984). *^13^C NMR Spectroskopie*.

[29] Ali S, Ahmad F, Mazhar M, Munir A, Masood MT (2002). Synthesis and spectral studies of di- and triorganotin(IV) complexes with 2-(6-methoxynaphthyl)propionic acid (naproxen). *Synthesis and Reactivity in Inorganic and Metal-Organic Chemistry*.

[30] Holeček J, Nádvorník M, Handlíř K, Lyčka A (1986). ^13^C and ^119^Sn NMR spectra of Di-*n*-butyltin(IV) compounds. *Journal of Organometallic Chemistry*.

[31] Williams DH, Fleming I (1987). *Spectroscopic Methods in Organic Chemistry*.

[32] Konstantinović SS, Radovanović BC, Sovilj SP, Stanojević S (2008). Antimicrobial activity of some isatin-3-thiosemicarbazone complexes. *Journal of the Serbian Chemical Society*.

[33] Singh RV, Chaudhary P, Chauhan S, Swami M (2009). Microwave-assisted synthesis, characterization and biological activities of organotin (IV) complexes with some thio schiff bases. *Spectrochimica Acta A*.

[34] Rehman W, Baloch MK, Badshah A, Ali S (2006). Synthesis characterization and biological study of diorganotin(IV) complexes of monomethyl phthalate. *Spectrochimica Acta A*.

[35] Chaturvedi KK, Goyal M (1984). Antibacterial studies of 7-(*α*-substituted sulphonamido)methyl- and 7-(*α*-substituted sulphonamido)phenyl-8-hydroxyquinolines. *Journal of the Indian Chemical Society*.

[36] Singh HL, Khungar B, Tripaathi UD, Varshney AK (2001). Spectral and antimicrobial studies of organotin(IV) complexes of bidentate schiff bases having nitrogen and sulphur donor systems. *Main Group Metal Chemistry*.

[37] Fritsche TR, McDermott PF, Shryock TR, Walker RD (2007). Agar dilution and disk diffusion susceptibility testing of *Campylobacter* spp.. *Journal of Clinical Microbiology*.

[38] Jabeen M, Ali S, Shahzadi S (2012). Homobimetallic complexes of ligand having O and S donor sites with same and different di- and trialkyl/aryltin(IV) moiety their synthesis, spectral characterization and biological activities. *Journal of Iranian Chemical Society*.

[39] Shahzadi S, Shahid K, Ali S, Bakhtiar M (2008). Characterization and antimicrobial activity of organotin(IV) complexes of 2-[(2′,6′-diethylphenylamido)]benzoates and 3-[(2′,6′-diethylphenylamido)]propanoates. *Turkish Journal of Chemistry*.

[40] Ashfaq M, Khan MI, Baloch MK, Malik A (2004). Biologically potent organotin(IV) complexes of 2-maleimidoacetic acid. *Journal of Organometallic Chemistry*.

